# Supplementary material for: A short plus long-amplicon based sequencing approach improves genomic coverage and variant detection in the SARS-CoV-2 genome
Source: PLoS One. 2022 Jan 13;17(1):e0261014. doi: 10.1371/journal.pone.0261014 (PMC8757904; doi:10.1371/journal.pone.0261014)
Supplement: Attachment — Submitted filename: Response to reviewers.docx [file pone.0261014.s009.docx]

PONE-D-21-19442
A Short Plus Long-Amplicon Based Sequencing Approach Improves Genomic Coverage and Variant Detection In the SARS-CoV-2 Genome
PLOS ONE

Authors thank all the reviewers for taking time to read our revised manuscript and provide valuable suggestions to further improve it. We have responded to each and every point raised by the reviewers. All the updates has been included in the revised manuscript.

Reviewer #1: all comments have been addressed in the revised version , this article may be accepted.

Response: We appreciate reviewer for taking time to review our revised manuscript.

Reviewer #2: The revised manuscript, "A Short Plus Long-Amplicon Based Sequencing Approach Improves Genomic Coverage and Variant Detection In the SARS-CoV-2 Genome" by Arana et al. has satisfactorily addressed all of my major comments and concerns. Aside from a couple of minor suggestions to improve the clarity of the figures (below), I think the data and interpretations are solid and support publication.
Response: We appreciate reviewer for taking time to keenly review our revised manuscript.

Minor points
-Figure 2: Harmonizing the labels (Assay I, Assay II, etc.) with those used in Figure 1 would help to make the figure more easily interpretable. Numbers are used for the assays in Figure 1 and 3, but roman numerals are used in Figure 2.

Response: Figure 2 has been updated as per the reviewer’s suggestion.

-Figure 6: Coverage values are rounded to the nearest integer. I suggest adding an additional decimal point as in Figure 2.

Response: Figure 6 has been updated as per the reviewer’s suggestion.
